# EthA/R-Independent Killing of *Mycobacterium tuberculosis* by Ethionamide

**DOI:** 10.3389/fmicb.2017.00710

**Published:** 2017-04-25

**Authors:** Michelle L. T. Ang, Siti Z. Zainul Rahim, Paola Florez de Sessions, Wenwei Lin, Vanessa Koh, Kevin Pethe, Martin L. Hibberd, Sylvie Alonso

**Affiliations:** ^1^Department of Microbiology and Immunology, Yong Loo Lin School of MedicineSingapore, Singapore; ^2^Immunology Programme, Life Sciences Institute, National University of SingaporeSingapore, Singapore; ^3^Genome Institute of SingaporeSingapore, Singapore; ^4^Lee Kong Chian School of Medicine and School of Biological Sciences, Nanyang Technological UniversitySingapore, Singapore; ^5^Department of Pathogen Molecular Biology, London School of Hygiene and Tropical MedicineLondon, UK

**Keywords:** ethionamide, *Mycobacterium tuberculosis*, multi-drug resistant tuberculosis, *mshA*, ethA/R locus

## Abstract

Ethionamide (ETH) is part of the drug arsenal available to treat multi-drug resistant tuberculosis. The current paradigm of this pro-drug activation involves the mycobacterial enzyme EthA and the transcriptional repressor, EthR. However, several lines of evidence suggest the involvement of additional players. The *ethA/R* locus was deleted in *Mycobacterium bovis* BCG and three *Mycobacterium tuberculosis* (MTB) strains. While complete resistance to ETH was observed with BCG *ethA/R* KO, drug susceptibility and dose-dependent killing were retained in the *ethA/R* KO MTB mutants, suggesting the existence of an alternative pathway of ETH bio-activation in MTB. We further demonstrated that this alternative pathway is EthR-independent, whereby re-introduction of *ethR* in *ethA/R* KO MTB did not lead to increased resistance to ETH. Consistently, *ethA* KO MTB (with intact *ethR* expression) displayed similar ETH susceptibility profile as their *ethA/R* KO counterparts. To identify the alternative ETH bio-activator, spontaneous ETH-resistant mutants were obtained from *ethA/R* KO MTB and whole genome sequencing identified single nucleotide polymorphisms in *mshA*, involved in mycothiol biosynthesis and previously linked to ETH resistance. Deletion of *mshA* in *ethA/R* KO MTB led to complete ETH resistance, supporting that the role of MshA in ETH killing is EthA/R-independent. Furthermore *mshA* single KO MTB displayed levels of ETH resistance similar or greater than those obtained with *ethA/R* KO strains, supporting that *mshA* is as critical as *ethA/R* for ETH killing efficacy.

## Introduction

Approximately one-third of the world population is presently infected with *Mycobacterium tuberculosis* (MTB), and this worldwide epidemic appears to be deteriorating. Underlying this endemic is the emerging epidemic of multi-drug resistant (MDR-TB) and extensively drug resistant (XDR-TB) TB strains that have severely undermined control efforts (WHO, 2016). With dwindling treatment options for MDR and XDR-TB that are decades old, one of the pertinent key issues faced by the TB research community is the daunting challenge of synthesizing new anti-TB drugs with novel modes of action (Koul et al., 2011). Since the discovery of Rifampicin (RIF) 40 years ago, few promising anti-TB drugs have been discovered, much less successfully entered the TB clinical pipeline (Singh and Mizrahi, 2016). The recent approval of bedaquiline (BDQ) (Andries et al., 2005; Diacon et al., 2014; Pym et al., 2016) and delamanid (Gupta et al., 2016) to treat MDR-TB represents a critical milestone. However, the emergence of clinical resistance less than 3 years after BDQ introduction to medical use is likely to limit the impact of this new TB drug (Bloemberg et al., 2015). Being further hampered by the unfavorable economics of TB drug development (Koul et al., 2011), the identification and commercialization of new anti-TB drugs may take up to another decade. In addition, more appropriate clinical trials to properly evaluate the efficacy of anti-TB drugs used in MDR and XDR-TB patient groups are necessary along with the improvement in TB diagnostics for a wider coverage of drug susceptibility testing (Koul et al., 2011).

Improving the efficacy of existing drugs may represent an alternative strategy of choice that should not be disregarded. This approach, however, necessitates further understanding in the mechanism of action of mycobacterial drugs and their bio-activation, especially drugs which have been suggested to have multiple targets and pathways, such as isoniazid (INH) (Vilchèze et al., 2006; Vilchèze and Jacobs, 2007) and ethionamide (ETH) (Morlock et al., 2003).

Despite its clinical use in humans for over 40 years since its first synthesis in 1956, ETH prescription has been restricted to patients infected with MDR-TB strains due to its associated side effects including serious hepatotoxicity, gastro-intestinal disturbances, and other adverse toxicity issues (Jenner and Smith, 1987). Consequently, this has led to poor patients’ compliance and unsatisfactory treatment outcome due to drug dosage that falls within the sub-optimal efficacy range. The recent discovery of small molecules capable of boosting ETH killing efficacy supports the idea that it is possible to improve ETH treatment through dosage reduction, thus minimizing side effects and improving patient compliance (Willand et al., 2009; Flipo et al., 2011). As the number of MDR-TB cases climbs, ETH has become an increasingly important second-line drug for the treatment of MDR-TB (Vale et al., 2013).

A structural analog of INH, ETH is a thioamide pro-drug that like INH, inhibits the molecular target InhA, a NADH specific enoyl-acyl carrier protein reductase, to eventually inhibit mycolic acid synthesis (Quemard et al., 1992; Banerjee et al., 1994). However, while both INH and ETH exert inhibitory actions on InhA, the pathways for pro-drug activation and mode of action toward their target are distinct (Zhang et al., 1992; Dessen et al., 1995; Rozwarski et al., 1998; Baulard et al., 2000). Several lines of experimental evidence point to the Baeyer–Villiger monooxygenase (BVMO) EthA as the mycobacterial enzyme responsible for ETH bio-activation (Baulard et al., 2000; DeBarber et al., 2000; Dover et al., 2007). However, the physiological role of EthA remains unknown although we previously reported that it is involved in the cell wall mycolic acids composition with consequence on the adherence properties of MTB to mammalian cells (Ang et al., 2014).

In addition to ETH, EthA is a bio-activator of other thiocarbamide-containing drugs, in particular thiacetazone (TAC) and isoxyl (ISO), suggesting broad substrate specificity for this enzyme (Dover et al., 2007). Like ETH, TAC, and ISO have been shown to target the mycolic acid biosynthesis, albeit through a different mode of action. The mechanism of action of TAC remains poorly understood, while ISO as well as its derivatives were shown to inhibit the synthesis of both fatty acids and mycolic acid subtypes (Bhowruth et al., 2006).

Specifically, ETH activation by EthA results in the production of various intermediates and derivative metabolites, among which the active major compound, ETH^∗^ has yet to be structurally identified (Vannelli et al., 2002; Hanoulle et al., 2005, 2006). High resolution magic angle spinning-NMR (HR-NMR) studies analyzed the distribution of ETH-derived metabolites inside and outside the bacteria, while monitoring the kinetics of the drug transformation (Hanoulle et al., 2005). ETH was found to be metabolized by EthA into ETH-SO and ETH^∗^, and subsequently into ETH-OH. Only ETH^∗^ was observed to accumulate within the bacterial cells whilst the other ETH derivatives were exclusively found in the extracellular milieu, pointing at ETH^∗^ as the prime active compound candidate for antibiotic action (Hanoulle et al., 2006). However, due to the technical challenges to purify ETH^∗^, its molecular characterization remains unknown.

**FIGURE 1:**
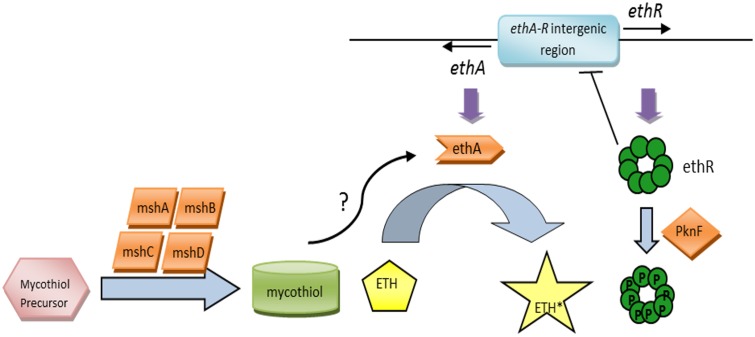
**The ETH bio-activation paradigm.** ETH is activated by the monooxygenase EthA into its activated form, ETH^∗^. The expression of EthA is regulated by the transcriptional repressor EthR, and both *ethA* and *ethR* are organized in a divergent operon with a shared intergenic promoter region. EthR dimers bind cooperatively as a homo-octamer to the specific operator in the *ethA-ethR* intergenic promoter region, repressing both *ethA* and *ethR* expression. The mycobacterial serine/threonine protein kinase PknF negatively regulates the physical binding of EthR to the DNA via phosphorylation of the EthR homo-octamer, hence promoting *ethA-ethR* expression. Additionally, the mycothiol synthesis pathway and its end product, mycothiol, have been implicated in ETH bio-activation as well.

Expression of EthA-encoding gene *ethA* is regulated by the product of its neighboring gene *ethR* (Baulard et al., 2000; DeBarber et al., 2000; Engohang-Ndong et al., 2004), with both *ethA* and *ethR* arranged in a divergent operon with a shared intergenic promoter region (**Figure 1**). EthR is a repressor that belongs to the TetR/CamR family of transcriptional regulators. Overexpression of *ethR* resulted in ETH resistance; whereas chromosomal inactivation of *ethR* led to ETH hypersensitivity, suggesting that EthR represses *ethA* expression in mycobacteria (Engohang-Ndong et al., 2004). EthR dimers bind cooperatively as a homo-octamer to the specific operator in the *ethA-ethR* intergenic promoter region, repressing the divergent transcription of both *ethA* and *ethR* (Engohang-Ndong et al., 2004). The X-ray crystal structure of EthR in a ligand-bound conformation (EthRHexOc) was reported and described as a homodimer with a ligand bound to each EthR monomer, the ligand subsequently being identified as hexadecyl octonoate (HexOc) (Frenois et al., 2004). In the presence of HexOc, EthRHexOc is unable to bind to its target DNA and thus fails to repress *ethA* transcription (Frenois et al., 2006). This has led to the identification of synthetic inhibitors of EthR that can improve by up to 10-fold ETH potency against MTB (Willand et al., 2009), thereby supporting that boosting ETH bio-activation represents an interesting approach to improve ETH killing potency without increasing dosage, thus improving the therapeutic index of ETH.

The identification of a fairly large proportion of ETH-resistant clinical isolates with no known genes linked to ETH resistance (Morlock et al., 2003) clearly suggests the existence of additional factors and pathways involved in ETH bio-activation and killing efficacy. The mycobacterial serine/threonine protein kinase PknF was identified to negatively regulate the DNA-binding activity of EthR via phosphorylation of the EthR homo-octamer, hence promoting *ethA-ethR* expression (Leiba et al., 2014) (**Figure 1**). This finding suggests that *ethA* gene expression is tightly regulated and involves more than one modulator. A few years ago, the glycosyltransferase MshA and its downstream product, mycothiol, the mycobacterial analog for glutathione have also been proposed to contribute to ETH bio-activation (Vilchèze et al., 2008, 2011) (**Figure 1**), although the detailed molecular mechanisms remain to be elucidated. More recently, another mycobacterial Baeyer–Villiger monooxygenase, MymA, was reported to be able to activate ETH, as evidenced by increased MIC_90_ of ETH in *mymA*-overexpressing MTB and increased resistance to ETH in loss-of-function *mymA* mutants (Grant et al., 2016). Even more recently, a cryptic alternative bio-activation pathway of ETH has been reported which consists of a bicistronic divergent operon (*rv0077c-rv0078*) sharing homologies with the *ethA/R* locus although no cross-talk between the two regulons could be demonstrated (Blondiaux et al., 2017).

In this work, we generated *ethA/R* KO BCG and MTB mutants and showed that while the BCG KO strain displayed complete resistance to ETH, drug susceptibility and dose-dependent killing were retained in the *ethA/R* KO MTB mutants, thus supporting the existence of a functional alternative pathway of ETH bio-activation in MTB that is independent of EthA/R. We further demonstrated that this alternative pathway is independent of the transcriptional repressor EthR. Generation of spontaneous ETH-resistant mutants confirmed a role for MshA in ETH killing activity in an EthA/R-independent manner.

## Materials and Methods

### *Escherichia coli* Growth Conditions

All *Escherichia coli* strains were grown in Luria-Bertani (LB) broth and agar (Difco). When appropriate, hygromycin and kanamycin were added at 150 and 50 μg/ml into the medium, respectively. Chemically competent *E. coli* TOP10 strain (Invitrogen) was used for propagation of all plasmids in this study.

### *M. bovis* BCG and MTB Strains, and Growth Conditions

*Mycobacterium bovis* BCG was purchased from ATCC (Pasteur strain ATCC 35734), while MTB Erdman, H37Rv and CDC1551 strains were a kind gift from Novartis Institute for Tropical Diseases (NITD), Singapore. All these strains and their derivative were grown at 37°C in Middlebrook 7H9 liquid media (Difco) supplemented with ADS (0.5% bovine serum albumin-fraction V, 0.2% dextrose, 0.085% saline) or with OADC (0.5% bovine serum albumin-fraction V, 0.2% dextrose, 0.085% saline, 0.005% oleic acid, and 0.0004% catalase) enrichment as indicated. For growth on agar, 7H11 (Difco) containing 0.05% Tween 80 (Tw) and 0.2% glycerol supplemented with OADC was used. Appropriate antibiotics [80 μg/ml hygromycin (Roche), 20 μg/ml kanamycin (Sigma)] were added when required.

### Construction of KO Mutants and Complemented Strains

The *ethA/R* locus was deleted by double homologous recombination as described previously (Bardarov et al., 2002). Briefly, ∼800 bp-long DNA regions flanking the *ethA/R* locus in *M. bovis* BCG and MTB strains were amplified by polymerase chain reaction (PCR) and cloned directionally into vector pYUB854 (Bardarov et al., 2002) with the hygromycin-resistance cassette (*hyg*) lying in between the flanking regions. The *lacZ* ORF and promoter region from the pGoal17 plasmid (Parish and Stoker, 2000) were then cloned into the unique *Pac*I site of the pYUB construct. To prepare electrocompetent mycobacteria, 10 mL cultures were grown to mid-log phase [optical density at 600 nm (OD_600_) of 0.4–0.6] in 7H9-ADS in the absence of glycerol, followed by culture in fresh 7H9-ADS supplemented with 1.5% glycine (Sigma) 1 day prior to electroporation. On the day of electroporation, mycobacteria cells were washed thrice with 0.05% Tween80 before final resuspension in 1 ml of the same medium. 200 μl of electrocompetent mycobacteria suspension were electroporated (2.5 kV, 800 Q, 25 mF) with 2 μg of treated recombinant plasmid subjected to ultraviolet (UV) treatment as previously reported (Parish and Stoker, 2000) and plated onto Hygromycin-containing 7H11 medium supplemented with 40 μg/ml X-gal. White hygromycin-resistant clones were selected after 16 days incubation at 37°C and screened by PCR with a set of internal *ethA/R* primers (5′-TCC AGC GGT TTT CCG CGG TC-3′ and 5′-TCC CGG TGC GCC ACA TGT TC-3′).

To complement the *ethA/R* KO mutants, the 2.2 kb *ethA/R* full-length locus was PCR amplified (Supplementary Table S1), cloned into the multiple cloning site of the integrative vector pMV306 (Stover et al., 1991) and introduced into the genome of all *ethA/R* KO mutants via electroporation as described above. The resulting transformants were plated onto kanamycin-containing 7H11 agar. After 16 days incubation at 37°C, kanamycin-resistant colonies were PCR screened using the internal *ethA/R* primers as mentioned above.

The *ethA* KO and *mshA* KO mutants were obtained by double homologous recombination as described above, whilst complement *ethR* and complement *mshA* mutants were constructed in selected MTB strains using vector pMV306. Overexpressing *ethR* mutants were constructed using the multicopy replicative plasmid pMV262 (Stover et al., 1991). All plasmid constructs were synthesized and validated using oligonucleotides listed in Supplementary Table S1.

Construction of the double *mshA/ethA/R (m/e)* double KO mutants was obtained by first unmarking the *ethA/R* KO mutants in order to remove the hygromycin cassette which was inserted in place of the *ethA/R* locus. To do so, the *ethA/R* KO mutants were transformed with plasmid pYUB870 which harbors a χδ-resolvase (tnpR)-encoding gene (Bardarov et al., 2002), thereby allowing resolvase-mediated cleavage of the hygromycin cassette. Gentamicin-resistant clones were first selected after incubation at 31°C for the resolvase activity and selected again on 7H11 containing 2% sucrose after incubation at 39°C. Loss of the hygromycin cassette and retained deletion of the *ethA/R* locus were verified by PCR. Successfully unmarked *ethA/R* KO mutants were then used for deletion of *mshA* by classical double homologous recombination as described above, followed by complementation of *mshA* under the *hsp60* promoter and using pMV306 plasmid (Supplementary Table S1).

### Southern Blot Analysis

1–3 μg of genomic DNA (gDNA) was digested with *Sac*I (Promega), separated on a 1.5% agarose gel and treated as previously reported (Sambrook and Russell, 2001). DNA was transferred onto a Millipore Immobilon-Ny+ Transfer membrane and UV cross-linked. For detection of *ethA/R* and *ethA* KO mutants, a 415 bp DIG-labeled probe was amplified using a set of primers that bind approximately 1.5 kb downstream of *ethR*, 5′-TGA GTT TAG TTG GGA CCT AGG CC-3′and 5′-CTA GAG TCA CAT CAG AAA CAT TTG A-3′. For detection of *mshA* and double *mshA/ethA/R* KO mutants, a 600 bp DIG-labeled probe was amplified using a set of primers that bind immediately upstream of *mshA*, 5′-CCC GTC CAC TCT GAA ATG CTC G-3′ and 5′-ATC AAC CCT GAA CCG TCA TCG TGT-3′. Probe amplifications were done via PCR according to the manufacturer’s instructions (DIG-labeling kit, Roche). Hybridization and signal detection were performed using a detection kit (Roche) according to the manufacturer’s protocol. EasyHyb (Roche) was used as the pre-hybridization and hybridization solutions, and CSPD (Roche) was used as the detection substrate for chemical luminescence.

### *In Vitro* Drug Susceptibility Assays

Bacterial drug susceptibility assays were performed in 7H9 media supplemented with either ADS or OADC (as indicated in the figure legends) as described previously (Kurabachew et al., 2008). ETH (Sigma), ISO (NITD), and TAC (NITD) were dissolved in 90% DMSO, whilst INH (Sigma) was dissolved in ultrapure water for stock solutions. Using a broth microdilution method, INH and ETH were twofold serially diluted (0.02–5 and 0.3–80 μM respectively) in 7H9 medium in 96-well flat bottom plates. Log phase MTB cultures were diluted in 7H9-OADC or 7H9-ADS medium to obtain ∼2 × 10^5^ colony forming units (CFUs)/ml. 100 μl of the bacterial inoculum were added to each well-containing an equal volume of drug suspension. The plates were then incubated for 5–7 days at 37°C. On the 5th or 7th day (as indicated), OD_600_ was measured using a Biorad iMark Microplate absorbance reader and curves were plotted using PRISM to determine the minimal inhibitory concentration (MIC_50_) values. The MIC_50_ is defined as the lowest concentration of drug that is required to inhibit 50% growth of the MTB strain compared to growth obtained in drug-free 7H9 medium. Drug assays were performed thrice independently. After determining the MIC_50_ values, 50 μl of the bacterial suspensions incubated at 1x, 2x, and 4x MIC_50_ were plated onto 7H11 agar plates. CFU were enumerated after 16 days incubation at 37°C and the CFU-based MIC_90_ range (in μM) was defined as the range of drug concentrations within which the number of CFUs compared to the drug free control was reduced by 90% (1 log reduction).

### Isolation of ETH-resistant Spontaneous Mutants

To generate spontaneous ETH^R^ clones from the Erdman *ethA/R* KO mutant, a protocol was adapted from both Luria and Delbrück (1943) and Mathys et al. (2009). Since the Erdman *ethA/R* KO mutant displays low resistance to ETH, optimization was performed to determine the appropriate ETH concentration range that would minimize background and false positive. Eventually, 360, 420, and 480 μM ETH were selected as appropriate ETH concentrations.

To generate spontaneous ETH^R^ mutants, an exponential phase 7H9-ADS liquid culture (OD_600_ of 0.6–0.8) of the *ethA/R* KO mutant was used to inoculate three individual flasks of 7H9-ADS medium at an initial OD_600_ of 0.005–0.01. The cultures were incubated for 1–2 weeks. An estimated 10^8^ and 10^7^ bacteria were then plated onto 7H11 plates containing selected ETH concentrations. ETH^R^ colonies were picked and grown in liquid medium for gDNA extraction and glycerol stocks. The MIC of ETH was determined for these clones, and extracted gDNA was used for whole genome sequencing (WGS).

### Whole Genome Sequencing (WGS) of ETH^R^ Mutants

#### Library Building

Two ug of gDNA was fragmented to a peak size range of 200–400 bp using Covaris S2 (Covaris, Woburn, MA, USA) (shearing conditions – Duty cycle: 20%; Intensity: 4; Cycles per burst: 200; Time: 360 s). The fragmented samples were then purified (Qiagen PCR purification kit; Qiagen, Valencia, CA, USA), and quality-checked (2100 Bioanalyzer on a DNA 1000 Chip, Agilent Technologies, Santa Clara, CA, USA). The whole genome library was prepared using Illumina’s TruSeq DNA Sample Preparation Kit, v2 (part number 15026486) according to the manufacturer’s instructions. Fragments in the range 300–500 bp were selected on a Pipen Prep from Sage Science, and quality-checked. Finally, using the Multiplexing Sample Preparation Oligonucleotide Kit (Illumina, San Diego, CA, USA), samples underwent 14 PCR cycles followed by Agencourt AMPure XP magnetic bead (Beckman Coulter, Brea, CA, USA) clean up according to the manufacturer’s instructions. qPCR was then performed using LightCycler 480 SYBR Green I Master mix (Roche Applied Science, Indianapolis, IN, USA) in a LightCycler^®^ 480 II real time thermal cycler (Roche Applied Science, Indianapolis, IN, USA) according to the manufacturer’s instructions.

#### Multiplexed Sequencing

Next generation sequencing (NGS) was done using Illumina Hiseq 2000 flow cell, 2 × 76 base pair-end runs. PhiX was used as control.

#### Analysis of Whole Genome Sequences

Unix Korn Shell^1^ was used to access the server and perform the quality control of raw reads (fastq files) and file transfer.

### Statistical Analysis

Unless otherwise stated, bars represent means + standard deviations (SD) and averages were compared using a bidirectional unpaired Student’s *t*-test with a 5% significance level (^∗^*p* ≤ 0.05).

## Results

### MTB Remains Susceptible to ETH Despite Removal of the *ethA/R* Locus

The *ethA/R* locus was removed and substituted in place by the hygromycin-resistant cassette *hyg* in *M. bovis* BCG and three MTB backgrounds, namely Erdman, H37Rv and CDC1551 (Supplementary Figure S1). The minimum inhibitory concentration of drug required to inhibit 50% growth of mycobacteria compared to drug-free control (MIC_50_) was measured for the respective *ethA/R* KO mutant strains in order to determine their level of resistance to ETH and to the two other thiocarbamide-containing drugs ISO and TAC. INH was included as well as a negative control since bio-activation of INH is not EthA/R-dependent. Consistently, all *ethA/R* KO mutants displayed parental susceptibility to INH (**Table 1**). In contrast, and as expected, removal of the *ethA/R* locus in *M. bovis* BCG led to complete resistance to ETH (**Table 1** and Supplementary Figure S2). However, complete resistance to ETH was not seen with the MTB *ethA/R* KO mutants whereby a dose-dependent killing could still be observed as evidenced by the sigmoidal MIC curves obtained (Supplementary Figure S2). The MIC_50_ values measured with the *ethA/R* KO MTB mutants were increased by 2–3X compared to their respective parental strains (**Table 1**). Furthermore, all the *ethA/R* KO mutant strains displayed increased resistance to TAC, with MIC_50_ values increasing by 2–8X (**Table 1**). As for ISO, with the exception of Erdman *ethA/R* KO mutant which was found slightly more resistant with a twofold increase in the MIC_50_ value compared to its parental counterpart, no significant changes in MIC_50_ were observed with the other mutant strains. Importantly, parental susceptibility to the three drugs was restored in each strain upon re-introduction of the *ethA/R* locus (**Table 1** and Supplementary Figure S2).

**Table 1:** MIC_50_ of ETH in BCG and *M. tuberculosis* parental and mutant strains.

Strain	INH	ETH	ISO	TAC
BCG	0.38	15.86	3.93	0.76
BCG *ethA/R* KO	0.35	NA	3.85	5.91
BCG *ethA/R* KO complement *ethA/R*	0.39	14.49	4.04	0.27
CDC1551	0.22	6.90	12.40	9.20
CDC1551 *ethA/R* KO	0.22	12.40	11.56	19.28
CDC1551 *ethA/R* KO complement *ethA/R*	0.20	4.77	14.00	6.42
Erdman	0.15	3.89	10.87	4.80
Erdman *ethA/R* KO	0.11	9.29	21.20	12.12
Erdman *ethA/R* KO complement *ethA/R*	0.12	3.25	13.75	2.07
H37Rv	0.20	3.30	13.08	2.0
H37Rv *ethA/R* KO	0.20	9.16	10.67	12.32
H37Rv *ethA/R* KO complement *ethA/R*	0.19	3.10	11.59	1.47


*MIC_*50*_ concentrations are indicated in μM. NA, not available due to complete ETH resistance.*

To confirm the MIC_50_ data obtained, the bacterial suspensions incubated with ETH concentrations that corresponded to 1x, 2x, and 4x MIC_50_ were plated for CFU enumeration. The ETH concentration range within which a 90% reduction of the bacteria load (also equivalent to 1 log) compared to drug-free control was determined and called CFU-based MIC_90_. Consistent with the MIC_50_ data, while BCG *ethA/R* KO displayed full resistance to ETH, dose-dependent killing was observed with all three MTB KO strains over the range of ETH concentrations assayed (Supplementary Figure S3). The MIC_90_ range of ETH on CDC1551 *ethA/R* KO mutant was increased by 8–16 fold compared to its parental and complemented counterparts (**Table 2**). In contrast, a mild twofold increase in ETH MIC_90_ was observed with *ethA/R* KO H37Rv and Erdman strains compared to their parental and complemented counterparts. Although these three MTB strains belong to the same Lineage 4 (Euro-American), H37Rv and Erdman are considered as laboratory strains whereas CDC1551 is regarded as a “clinical” strain. The extensive passages *in vitro* are likely to result in genetic changes that may lead to distinct phenotypes both *in vitro* and *in vivo* when compared to CDC1551, including the drug susceptibility profile (Betts et al., 2000; Coscolla and Gagneux, 2010).

**Table 2:** CFU-based MIC_90_ of ETH in BCG and *M. tuberculosis* parental and mutant strains.

	Parental strain	*ethA/R* KO	*ethA/R* KO complement *ethA/R*	pMV262-*ethR*	pMV306-*ethR*	*ethA* KO
BCG	10–20	NA	10–20	ND	ND	ND
CDC1551	2.5–5	40	2.5–5	20–40	ND	20–40
Erdman	1.25–2.5	2.5–5	1.25–2.5	1.25–2.5	2.5–5	1.25–2.5
H37Rv	1.25–2.5	2.5–5	1.25–2.5	2.5–5	ND	2.5–5


*Bacterial suspensions corresponding to 1x, 2x, and 4x MIC_*50*_ were plated onto 7H11 agar plates. CFU were enumerated after 16 days incubation at 37°C and the CFU-based MIC_*90*_ range (in μM) was determined. pMV262-*ethR* was introduced into WT MTB strains. pMV306-*ethR* was introduced into *ethA/R* KO Erdman strain.*

*ND, not determined; DF, drug-free; NA, not available due to complete resistance to the drug.*

Altogether, these data show that although the ETH MICs values obtained for *ethA/R* KO MTB strains were higher than those measured with the corresponding parental and complemented strains, ETH susceptibility and dose-dependent drug response to ETH were retained. In particular both MTB Erdman and H37Rv strains remained very susceptible to ETH upon deletion of *ethA/R* locus with 3- and 2-fold increases in their MIC values respectively. The retained susceptibility to ETH despite removal of *ethA/R* in the three MTB strains suggests that the pro-drug ETH still gets activated into its bactericidal form in an EthA-independent manner, thus supporting the existence of an alternative bio-activation pathway for ETH in MTB.

### The Alternative Pathway of ETH Bio-activation in MTB Is Not under the Control of the Transcriptional Repressor EthR

To further investigate the possible existence of an alternative pathway of ETH bio-activation in MTB, we questioned whether the transcriptional repressor EthR which negatively modulates the *ethA/R* locus, would also modulate this alternative pathway. Indeed, using programs available online^2^, EthR was predicted to bind to a number of promoter regions in addition to the *ethA/R* intergenic region. Thus it is conceivable that in WT MTB, EthR may repress the expression of another gene that is involved in ETH bio-activation. To address this hypothesis, the *ethR* open reading frame (ORF) was over-expressed in all three WT MTB strains under the control of the constitutive strong promoter *hsp60* and using the multicopy replicative plasmid pMV262. Real-time PCR analysis confirmed the over-expression of *ethR* (8–16 fold increase) in comparison to the parental strains. Over-expression of *ethR* in these three strains was expected to lead to the strong repression of *ethA* as well as any other genes that may be negatively regulated by EthR. Therefore, should an alternative EthR-dependent pathway of ETH bio-activation exist in MTB, susceptibility to ETH would be affected when *ethR* is over-expressed. However, the *ethR* over-expressing MTB strains retained ETH susceptibility in a dose-dependent manner (Supplementary Figures S4A–C) and displayed CFU-based MIC_90_ values similar to those obtained with the *ethA/R* KO mutants (**Table 2**). These results thus support the existence of an alternative pathway of ETH bio-activation in MTB and indicate that this pathway is likely to be EthR-independent.

To confirm this observation, *ethA* single KO mutants were generated in the three MTB backgrounds. We reasoned that should EthR negatively repress the alternative ETH bio-activation pathway, *ethA* KO mutants would display full or increased resistance to ETH compared to their corresponding *ethA/R* KO counterparts. Results indicated that the CFU-based MIC_90_ ranges obtained with *ethA* single KO mutants were similar to the values obtained with the *ethA/R* KO mutants (**Table 2**), thus further supporting that the alternative pathway of ETH bio-activation is not negatively regulated by EthR.

Finally, *ethR* was re-introduced into the Erdman *ethA/R* KO mutant using integrative plasmid pMV306. We postulated that expression of *ethR* in the *ethA/R* KO mutant would only impact the susceptibility to ETH if the alternative pathway of ETH bio-activation is negatively regulated by EthR. However, ETH susceptibility was retained in this strain (Supplementary Figure S4D) and the MIC_90_ range was similar to that obtained with the *ethA/R* KO counterpart (**Table 2**).

Altogether, these findings strongly support the existence of an EthA/R-independent alternative pathway of ETH bio-activation in MTB strains.

### ETH^R^ Spontaneous Mutants Display Mutations in the *mshA* Locus

In order to identify the molecular players that are involved in the alternative pathway of ETH bio-activation in MTB, spontaneous mutants that were highly resistant to ETH were generated from the Erdman *ethA/R* KO mutant. Briefly, Erdman *ethA/R* KO bacteria were plated onto agar plates containing a range of ETH concentrations (360–480 μM). Two independent rounds of ETH^R^ mutants were generated. Individual ETH-resistant (ETH^R^) colonies were picked and sub-cultured. Drug susceptibility assays were then carried out for these ETH^R^ clones to confirm ETH resistance. No MIC_50_ values could be derived for these mutants as all mutants grew uninhibitedly even at the highest concentration of ETH (80 μM) tested (data not shown).

Since InhA is the downstream target of both ETH and INH (Banerjee et al., 1994; Vilchèze et al., 2005), a pre-screen was first conducted to exclude all the ETH^R^ spontaneous mutants harboring mutation(s) in the *inhA* gene. Previous literature has shown that the INH MICs obtained with most MTB isolates harboring mutations in *inhA* promoter region and ORF were usually low (>1 μg/ml) (Ramaswamy et al., 2003); hence, susceptibility to INH was assayed by growing the ETH^R^ clones in the presence of INH at 1 μg/ml (or 7.29 μM) and turbidity was visually assessed after 10 days incubation. For the great majority of the clones (∼90%) obvious turbidity was observed, indicating that these clones were INH^R^ thereby suggesting the presence of a mutation in their *inhA* locus. However, seven clones displayed impaired growth in the presence of 7.29 μM INH and further independent drug susceptibility assays indicated that these clones displayed varying levels of resistance to INH with MIC_50_ values ranging between 0.625 and 1.25 μM, with the exception of one clone displaying an unusually high level of INH resistance (5 μM). Sequencing revealed that there was no mutation in the *inhA* ORF and promoter region for each of the seven clones analyzed (data not shown).

The seven ETH^R^ clones were then subjected to WGS and compared against the Erdman *ethA/R* KO parental genome. Numerous mutations came up upon WGS, but the list was restricted to insertion/deletions (INDELs) and non-synonymous SNPs (NS-SNPs), and further refined by eliminating NS-SNPs that resulted in conservative amino acid changes (i.e., change to an amino acid with similar physiochemical properties). Strikingly, NS-SNPs were found in *mshA* for six out of the seven clones analyzed (Supplementary Table S2). Apart from *mshA*, mutations were found in other genes candidates as well, a large proportion of which are involved in metabolism pathways (*galE3, cobD, plcB*, and *pks5*). Another group of gene candidates included *gltx*, *recD*, and *topA* involved in the transcriptional, translational, and nucleotide assembly pathways (Supplementary Table S2). The remaining identified genes Erdman_1484 and Erdman_0263 could not be categorized under any pathways and these genes remained unclassified.

Since six out of the seven clones analyzed by WGS displayed a mutation in the *mshA* locus, we decided to further investigate the contribution of this gene in ETH bio-activation. MshA is a glycosyltransferase involved in mycothiol biosynthesis, an equivalent to the antioxidant glutathione in mammals, and was found to be essential for ETH susceptibility in MTB (Vilchèze et al., 2008). Furthermore, *mshA* mutations were shown to confer varying levels of co-resistance to INH and ETH in *M. bovis* (Vilchèze et al., 2011). Consistently, a recent study reported that 45.6% of ETH^R^ MDR-TB isolates harbored a mutation in *mshA* (Rueda et al., 2015). However, the role of the glycosyltransferase MshA in ETH-mediated killing has yet to be deciphered. The identification of ETH^R^ *ethA/R* KO Erdman mutants with mutations in *mshA* here further supports the role of MshA in ETH killing efficacy and suggests that it may be in an *ethA/R*-independent fashion.

### Construction and Growth Kinetic of MshA-deficient Mutants

To validate the role of MshA in ETH bio-activation, the *mshA* locus was deleted from WT MTB Erdman, H37Rv and CDC1551 strains, thus generating *mshA* single KO mutants. Furthermore, *mshA* was also deleted in the corresponding *ethA/R* KO mutants, giving rise to *mshA ethA/R (m/e)* double KO mutants. All clones were verified by Southern blot (Supplementary Figure S5) and subsequently complemented by introducing the *hsp60-mshA* construct into the mycobacterial genome using the integrative plasmid pMV306. Our choice to express *mshA* under *hsp60* promoter was driven by the fact that the *mshA* promoter has yet to be characterized and delineated.

**FIGURE 2:**
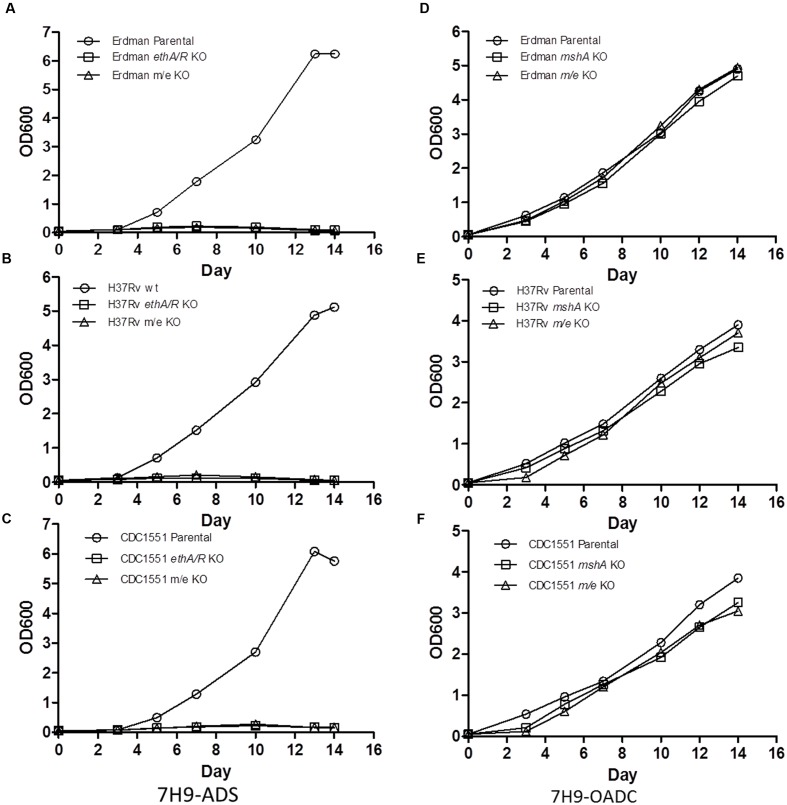
***In vitro* growth kinetics of *mshA* KO and *mshA ethA/R (m/e)* double KO mutants.** Growth kinetics of Erdman **(A,D)**, H37Rv **(B,E)**, CDC1551 **(C,F)** parental, *mshA* KO and *mshA/ethA/R (m/e)* double KO strains in 7H9 media supplemented with ADS **(A–C)** or OADC **(D–F)** over a period of 14 days. Every 2–3 days, OD_600_ was measured. The experiment was repeated twice independently.

Growth defect was previously reported for MshA-deficient MTB mutants (Vilchèze et al., 2008). Consistently, *mshA* and *m/e* KO mutants displayed a significant growth defect in 7H9 supplemented with ADS (**Figures 2A–C**). In contrast, all the KO strains grew well in 7H9-OADC (**Figures 2D–F**). However, it is worth to note that *mshA* and *m/e* KO mutants in the CDC1551 background still displayed a slight but significant and reproducible growth defect compared to their respective parental counterpart (**Figure 2F**).

Given that *mshA* and *m/e* KO mutants are unable to grow on 7H9-ADS, drug susceptibility assays involving these mutants were performed in 7H9-OADC. However, since the initial MIC values obtained in this work were generated in 7H9-ADS (Supplementary Figure S2), we re-established their MIC in 7H9-OADC. The MIC_50_ values obtained for INH were comparable regardless of the medium supplement (OADC or ADS) for the three MTB backgrounds (WT and *ethA/R* KO) (Supplementary Table S3). Similar MIC values for ETH were also observed with the three WT MTB strains in both types of medium. However, the ETH MICs increased by twofold for all *ethA/R* KO mutants upon replacing ADS with OADC (Supplementary Table S3). Despite the greater resistance to ETH in the presence of OADC, dose-dependent drug susceptibility to ETH was nevertheless retained (data not shown). Therefore, our data indicated that although some variation exists between the MIC values when using ADS or OADC as supplement, ETH susceptibility was still retained in the *ethA/R* KO MTB mutants.

### *mshA* Deletion Leads to ETH Resistance

Drug susceptibility assays were conducted with the *mshA* single KO and *mshA ethA/R* (*m/e*) double KO mutants. The slower growth rates observed with the CDC1551 *mshA* and *m/e* KO mutants (**Figure 2F**) were taken into consideration by reading the plates at day 7 post-setup.

Remarkably, the combined absence of both *ethA/R* and *mshA* loci in all three MTB backgrounds abrogated ETH susceptibility, rendering the *m/e* double KO mutants completely resistant to ETH (Supplementary Figure S6 and Table 3). MIC_50_ values could not be obtained for these mutants since mycobacteria grew uninhibitedly even at the highest concentration of ETH (80 μM) tested (**Table 3**). These observations were supported by the lack of a dose-response curve with all three *m/e* KO mutant strains (Supplementary Figure S6). Re-introduction of *mshA* in the *m/e* KO mutants restored ETH susceptibility to levels similar to those observed with their respective *ethA/R* KO counterparts (**Table 3**). Thus, these data indicate that the combined removal of *mshA* and *ethA/R* loci in MTB leads to complete resistance to ETH, and further confirms the involvement of *mshA* in ETH killing efficacy.

**Table 3:** MIC_50_ of INH and ETH on *mshA* single KO and *mshA ethA/R* double KO mutants.

Strain	MIC_50_ INH	MIC_50_ ETH
CDC1551	0.26	7.18
CDC1551 *ethA/R* KO	0.2	31.04
CDC1551 *ethA/R* KO complement *ethA/R*	0.29	5.12
CDC1551 *mshA* KO	0.98	NA
CDC1551 *mshA* KO complement *mshA*	0.43	11.82
CDC1551 *m/e* KO	0.57	NA
CDC1551 *m/e* KO complement *m/e*	0.30	4.76
Erdman	0.18	3.04
Erdman *ethA/R* KO	0.18	19.69
Erdman *ethA/R* KO complement *ethA/R*	0.17	2.67
Erdman *mshA* KO	0.49	20.63
Erdman *mshA* complement *mshA*	0.28	5.42
Erdman *m/e* KO	0.37	NA
Erdman *m/e* KO complement *m/e*	0.20	13.54
H37Rv	0.20	3.30
H37Rv *ethA/R* KO	0.28	12.55
H37Rv *ethA/R* KO complement *ethA/R*	0.23	1.99
H37Rv *mshA* KO	0.85	40.67
H37Rv *mshA* KO complement *mshA*	0.28	12.89
H37Rv *m/e* KO	0.80	NA
H37Rv *m/e* KO complement *m/e*	0.27	11.12


*Drug susceptibility assays were performed in 7H9-OADC. MIC_*50*_ are indicated in μM. NA, not available due to complete ETH resistance.*

Interestingly, deletion of *mshA* alone led to MIC_50_ values either comparable (Erdman background) or greater (H37Rv and CDC1551 backgrounds) than those obtained with their *ethA/R* KO counterparts (Supplementary Figure S6 and Table 3). This observation thus suggests that *mshA* is at least as critical as *ethA/R* for ETH killing efficacy. Complementation with *mshA* only partially restored the levels of ETH susceptibility, possibly due to the usage of *hsp60* promoter in place of its native promoter.

Additionally, and consistent with previous report (Vilchèze et al., 2008, 2011), the *mshA* KO mutants displayed mild increased resistance to INH with a twofold (Erdman) and fourfold (H37Rv and CDC1551) increase of the MIC_50_ values compared to the parental strains (**Table 3**).

Together, these data confirm the contribution of *mshA* in ETH and (to a lower extent) INH killing efficacy in MTB. In addition, the complete resistance to ETH upon deletion of *mshA* from *ethA/R* KO mutants suggests that the role of MshA in ETH killing efficacy is independent on EthA/R-mediated ETH bio-activation.

## Discussion

Previous studies had provided indirect experimental evidence of the involvement of EthA/R in ETH bio-activation either through over-expression of *ethA* or *ethR*, or through *ethR* deletion in *M. bovis* BCG (Baulard et al., 2000; DeBarber et al., 2000). Here, we show for the first time that deletion of the entire *ethA/R* locus in BCG led to full resistance to ETH, thus demonstrating the critical role of *ethA/R* in ETH bio-activation in BCG. However, deletion of *ethA/R* in MTB strains led to a mild increase only in their levels of resistance to ETH. In fact, susceptibility and dose-dependent drug response to ETH were retained in these *ethA/R* KO MTB mutants. Previous studies involving anti-mycobacterial pro-drugs INH and PZA have shown that absence of their respective bio-activators in MTB led to extremely high to complete levels of resistance (Heym et al., 1993; Morlock et al., 2000; Ng et al., 2004). The reported MICs of INH for *katG-*deleted mutants and KatG-deficient MTB isolates (∼80 mg/ml) (Heym et al., 1993) were 400-fold higher than the MIC measured with their WT and complemented counterparts (∼0.02 mg/ml), proving that deletion of *katG* is sufficient to confer high level of resistance to INH (Ng et al., 2004). Similarly, PZA-resistant strains with mutations in *pncA* that led to a loss in pyrazinamidase activity also displayed high levels of resistance to PZA, ranging from 100 to more than 800 μg/ml versus 12.5 μg/ml in WT counterparts (Morlock et al., 2000). Arguably, in the absence of their respective enzymatic bio-activator to convert these pro-drugs into a catalytically active form, these stable and chemically inert drug forms are expected to remain inactive and non-bactericidal, thus accounting for the high to complete levels of drug resistance observed. Previous studies by Hanoulle et al. (2006) have shown that ETH is metabolized into an ETH-S-oxide derivate (ETH-SO) and ETH^∗^, and subsequently into ETH-OH; out of which only ETH^∗^ was observed to accumulate within the bacterial cells. On the other hand, ETH, ETH-SO and ETH-OH were found exclusively in the extracellular milieu, suggesting ETH^∗^ to be the prime active compound candidate for antibiotic action. Other than ETH^∗^, all other ETH derivatives including pro-drug ETH itself possess little or no anti-mycobactericidal activity (Baulard et al., 2000; DeBarber et al., 2000; Vannelli et al., 2002; Francois et al., 2009). Here, the fact that deletion of the *ethA/R* locus did not lead to high level of resistance to ETH in MTB challenges the paradigm according to which *ethA/R* locus is solely responsible for ETH bio-activation in MTB (Baulard et al., 2000; DeBarber et al., 2000) and led us to propose the existence of a functional alternative pathway of ETH bio-activation independent of the *ethA/R* locus.

This alternative pathway of ETH bio-activation that is functional in MTB but not in BCG is likely to be distinct from the cryptic operon (*rv0077c-rv0078*) recently reported by Blondiaux et al. (2017), which is only expressed in the presence of small molecules that inhibit the EthR-like repressor (Rv0078), thereby allowing expression of *ethA2* gene (*rv0077c*) which encodes an EthA-like monooxigenase capable of activating ETH.

In fact, the genome of MTB encodes more than 30 putative monooxygenases which may have stemmed from evolution as a protective mechanism against various xenobiotic substances, leading Morlock et al. (2003) to propose the existence of one or more enzymes with functional redundancy to EthA. Consistently, five other putative BVMO-encoding genes have been previously reported in mycobacteria (Fraaije et al., 2004; Bonsor et al., 2006). The broad substrate specificity for BMVOs such as EthA (Fraaije et al., 2004) may support that one or more of these enzymes are capable of compensating for the loss of EthA for ETH activation function in MTB. Consistently, a recent study reported a role for BVMO MymA (Rv3083) in ETH activation (Grant et al., 2016). It is thus possible that the retained susceptibility to ETH observed in the MTB *ethA/R* KO strains is due to the presence of MymA, whereas MymA in BCG would be non-functional. A BLAST analysis^3^ indeed revealed two NS-SNPs in *mymA (Rv3083)* (I81A and V94I) between the MTB strains and BCG. However, both NS-SNPs map outside the ‘Baeyer–Villigerase’ (BVase) motif which is involved in the enzymatic activity of the protein (Bonsor et al., 2006). Therefore, we believe that these NS-SNPs are unlikely to account for a possible difference in the enzymatic activity of MymA between MTB and BCG. Further study is required in order to test the possible involvement of MymA in the differential susceptibility to ETH in BCG and MTB *ethA/R* KO mutants.

Among ETH^R^ MTB clinical isolates that have been reported, up to 20–50% harbor no mutations in genes known to be involved in ETH resistance (Morlock et al., 2003), adverting that the mechanisms involved in ETH killing efficacy involve additional players. To identify the mycobacterial factors involved in ETH bio-activation, we generated spontaneous ETH^R^ mutants from the *ethA/R* KO Erdman strain. Among the different gene candidates identified by WGS, *mshA* came out in six out of the seven clones analyzed. *mshA* mutations have been associated to resistance to INH (Jagielski et al., 2014, 2015) and ETH (Rueda et al., 2015) in clinical isolates. In addition, *mshA* mutations were shown to confer varying levels of co-resistance to INH and ETH in MTB (Vilchèze et al., 2008). Previous work has suggested that mycothiol, the final product of MshA pathway, plays a role in the EthA-mediated bio-activation steps of ETH (Vilchèze et al., 2008). Here, we demonstrated that removal of *mshA* in *ethA/R* KO MTB led to full resistance to ETH, thereby suggesting that the role of mycothiol in ETH killing efficacy is independent of, or at least not limited to, its interaction with EthA and may also be involved in the alternative pathway of ETH bio-activation, perhaps by interacting with the alternative BVMO MymA for example (Grant et al., 2016). Alternatively, MshA and mycothiol could play a role in ETH killing efficacy *downstream* ETH bio-activation. The fact that *mshA* deletion/mutations also affect the susceptibility to INH supports this latter hypothesis, since ETH and INH target the same molecule, InhA, through a comparable mechanism via the formation of a NAD-drug (INH/ETH) adduct. It is thus plausible that MshA activity plays a role during one of these common steps. We propose that mycothiol may either stabilize the formation of ETH^∗^ or form a complex with ETH^∗^. Additional experiments are necessary to further decipher the role of mycothiol in this process. Previous studies found that EthA is able to metabolize thiacetazone into either a sulfenic acid intermediate under acidic/neutral conditions or a carbodiimide metabolite under basic conditions; and both metabolites readily react with glutathione (GSH) to either regenerate the parent drug or form a GSH-adduct, respectively (Qian and Ortiz de Montellano, 2006). Since mycothiol is the mycobacterial analog for GSH, and EthA has also been shown to oxidize ETH into a sulfenic acid metabolite (Vannelli et al., 2002), ETH metabolites could react with mycothiol in a similar manner. Consequently, one could further speculate that such a reaction would either stabilize these reactive ETH metabolites or help in drug recycling, or perhaps lower the intracellular concentration of mycothiol thus sensitizing mycobacteria to oxidative damage, or culminate in a combination of all three consequences.

Whole genome sequencing analysis of the ETH^R^ spontaneous mutants has identified several other genes that may deserve further investigation. Of particular interest, Erdman_1484, a gene encoding for thioredoxin was identified. Interestingly, in *Streptomyces coelicolour*, the amount of mycothiol has been shown to be under the control of a sigma factor σ^R^, which is regulated by an anti-sigma factor RsrA via a thiol-disulphide redox switch involving thioredoxin (Park and Roe, 2008); One could speculate that such thiol-disulphide redox switch exists in mycobacteria as well, which could affect mycothiol levels, subsequently influencing the susceptibility to ETH killing. Moreover, since mycobacterial thioredoxins have been demonstrated to serve regulatory functions as disulphide reductants that affect the metabolism of mycobacteria (Van Laer et al., 2012; Machová et al., 2014), characterization of this gene may provide further insights into ETH killing efficacy. Additionally, since small thiol molecules do not appear to be directly associated with the thioredoxin system in bacteria unlike that in mammalian cells (Gustafsson et al., 2012), one could also speculate that thioredoxin may facilitate redox reactions specifically involved in the alternative pathway of ETH bio-activation.

## Conclusion

The data generated in this work provides the experimental evidence of the existence of a functional alternative pathway of ETH bio-activation in MTB that is independent of the *ethA/R* locus. Furthermore, they confirm the importance of MshA pathway and mycothiol in ETH killing efficacy, and may suggest a role of this pathway at a later step, after formation of the ETH cidal metabolite, ETH^∗^. Hence, together with the recent discovery of an alternative BVMO (MymA) capable of activating ETH (Grant et al., 2016), and a cryptic alternative pathway of ETH bio-activation (Blondiaux et al., 2017), our findings underscore the complexity and possible redundancy of the mechanisms involved in ETH bio-activation in pathogenic mycobacteria. Greater understanding of these mechanisms and their possible cross-talks are necessary in order to rationally design intervention strategies to improve ETH killing efficacy.

## Author Contributions

MA, SZ, PdS, WL, and VK performed the experiments; MA, SZ, PdS, KP, MH, and SA analyzed the data; MA, PdS, and SA wrote the manuscript. All authors approved the version to be published in Frontiers in Microbiology and agreed to be accountable for all aspects of the work.

## Conflict of Interest Statement

The authors declare that the research was conducted in the absence of any commercial or financial relationships that could be construed as a potential conflict of interest.

## Footnotes

1 http://www.kornshell.com/doc/

2 http://genome.tbdb.org/tbdb_sysbio/Resources.html

3 http://blast.ncbi.nlm.nih.gov/Blast.cgi
