# Supplementary material for: EthA/R-Independent Killing of Mycobacterium tuberculosis by Ethionamide
Source: Front Microbiol. 2017 Apr 25;8:710. doi: 10.3389/fmicb.2017.00710 (PMC5403819; doi:10.3389/fmicb.2017.00710)
Supplement: Supplementary file 1 [file Table_1.doc]

**Supplemental Information**

**EthA/R-independent killing of *Mycobacterium tuberculosis* by ethionamide**

**Running Title:** EthA/R-independent killing by ethionamide

**Authors:** Michelle Lay Teng Ang^1,2^, Siti Zarina Zainul Rahim^1,2^, Paola Florez de Sessions^3^, Wenwei Lin^1,2^, Vanessa Koh^1,2^, Kevin Pethe^4^, Martin Lloyd Hibberd^3,5^ and Sylvie Alonso^1,2*^.

**Affiliations:** ^1^Department of Microbiology and Immunology, Yong Loo Lin School of Medicine, ^2^Immunology programme, Life Sciences Institute, National University of Singapore, ^3^Genome Institute of Singapore, ^4^Lee Kong Chian School of Medicine and School of Biological Sciences, Nanyang Technological University, Singapore, ^5^Department of Pathogen Molecular Biology, London School of Hygiene & Tropical Medicine, UK.

*Correspondence:

Sylvie Alonso

[micas@nus.edu.sg](mailto:micas@nus.edu.sg); Fax: +65 67782684.

**
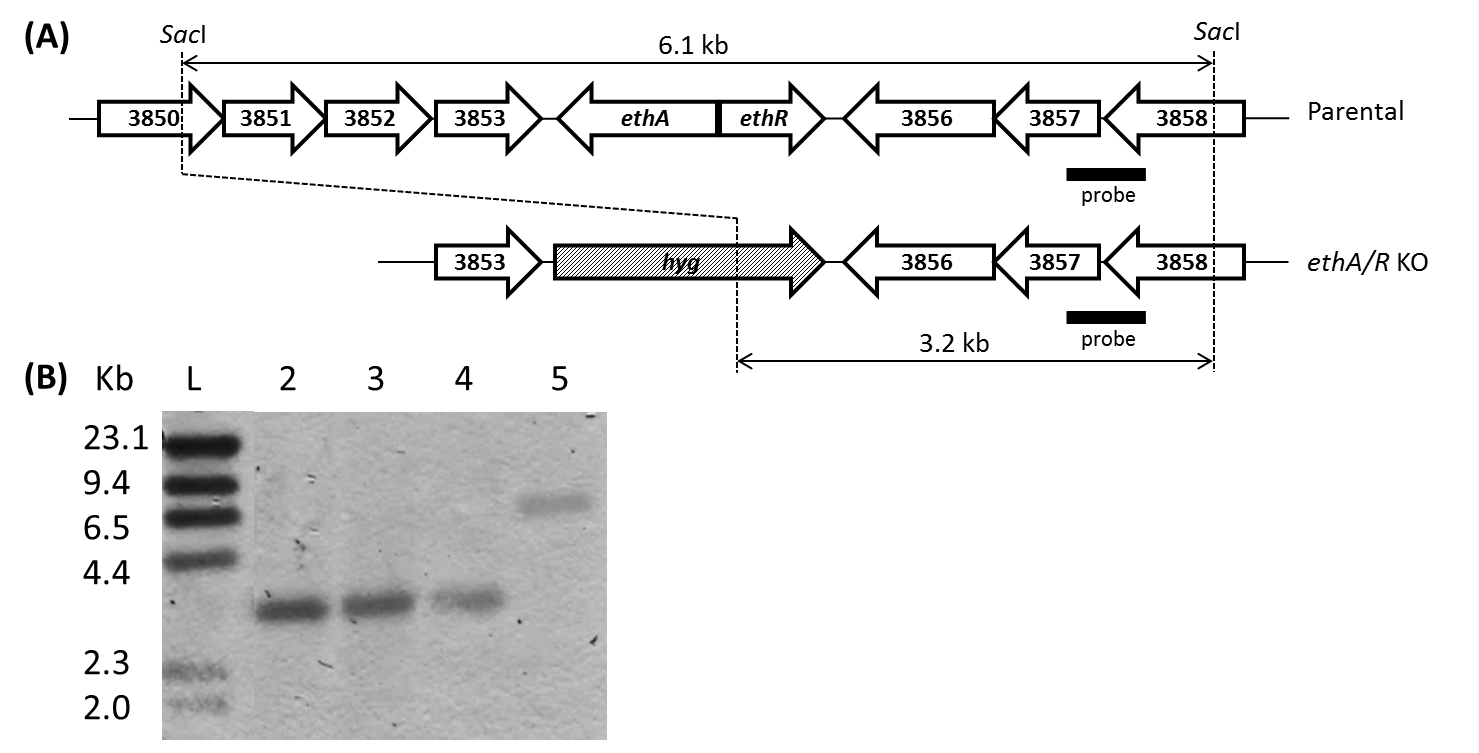
**

**Figure S1: Construction of *ethA/R* KO mutants in BCG, MTB Erdman, H37Rv and CDC1551.**

A) Chromosomal organization of *ethA/R* is identical in BCG and the three MTB backgrounds. The arrows depict the lengths and directions of *ethA*, *ethR* and their neighbouring genes. Black bar corresponds to the probe used for Southern Blot analysis. B) Southern Blot analysis of chromosomal DNA. L, DNA Molecular Ladder; 1, BCG *ethA/R* KO; 2, MTB Erdman *ethA/R* KO; 3, MTB H37Rv *ethA/R* KO; 4, MTB CDC1551 *ethA/R* KO ; 5, BCG WT.


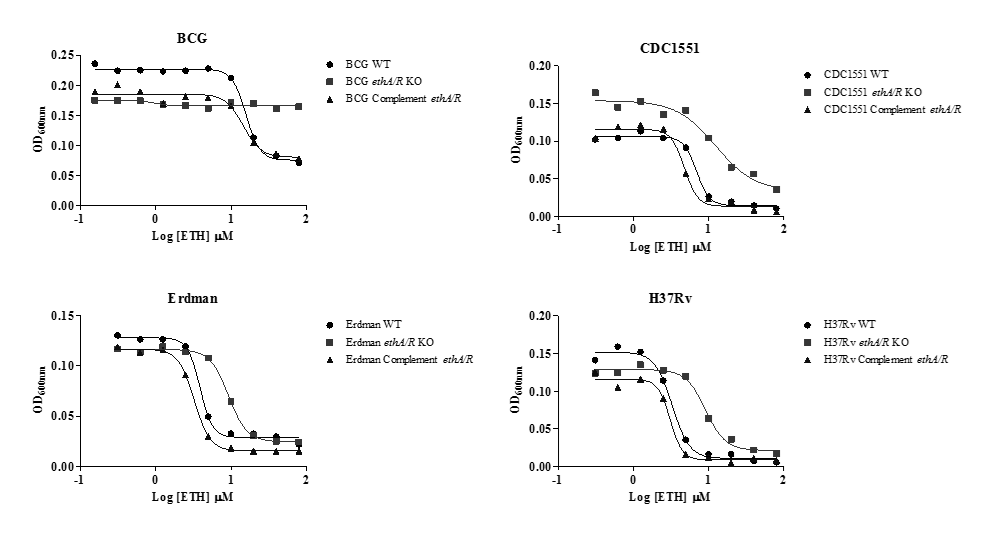


**Figure S2:** **Minimum Inhibitory Concentrations (MIC) of Ethionamide (ETH) and other drugs on *ethA/R* KO mutants.**

The assay was performed in 7H9-ADS. OD600 values were measured after 5 days incubation at 37^o^C. “BCG/CDC1551/Erdman/H37Rv complement” refers to *ethA/R* KO BCG/CDC1551/Erdman/H37Rv complemented with *ethA/R*.


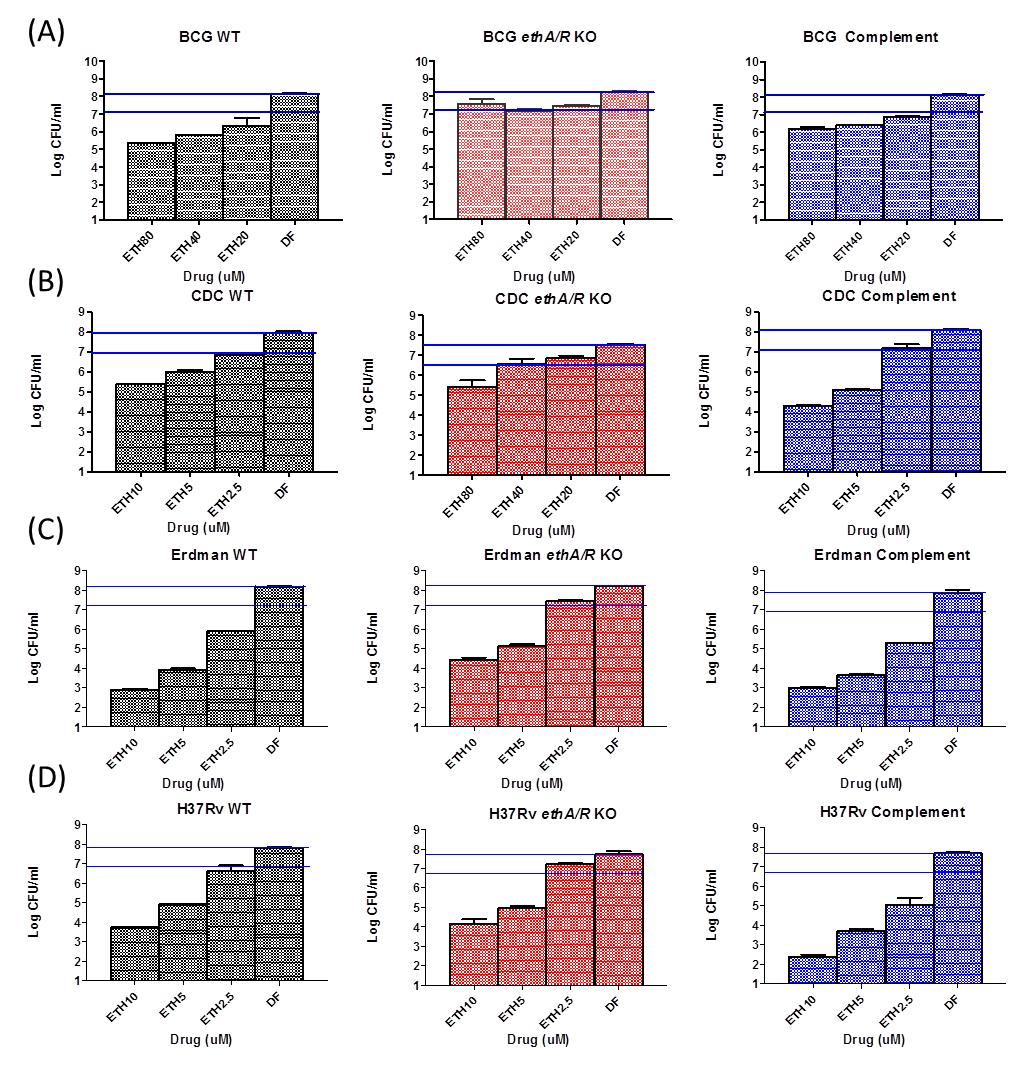


**Figure S3: CFU-based MIC_90_ of ETH on *ethA/R* KO mutants.**

BCG (A) or MTB (B-D) suspensions were incubated for 5 days in the presence of the indicated ETH concentrations and then plated onto 7H11 agar for colony counting after 16 days incubation. Horizontal lines represent 1 log reduction in CFU compared to the drug-free (DF) control. “BCG/CDC1551/Erdman/H37Rv complement” refers to *ethA/R* KO BCG/CDC1551/Erdman/H37Rv complemented with *ethA/R*.


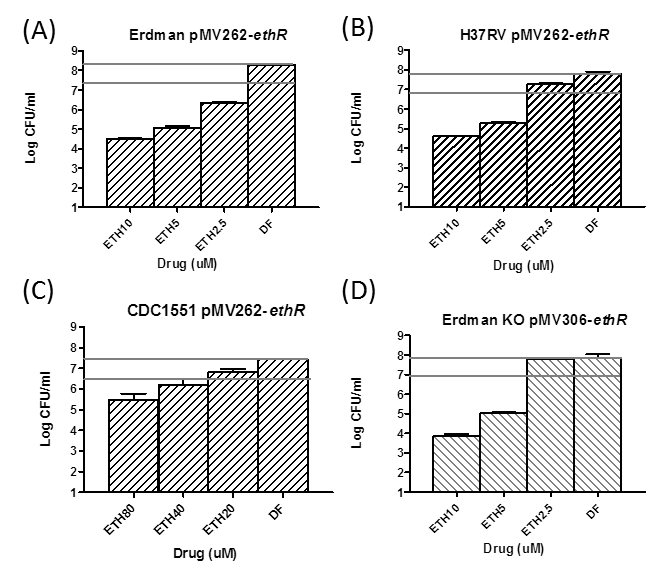


**Figure S4: CFU-based MIC_90_ of ETH on *ethA/R* KO mutants expressing *ethR*.**

Same legend as in Figure S3. *EthR* was over-expressed (pMV262-*ethR* construct) in Erdman (A), H37Rv (B) and CDC1551 (C) WT strains. *EthR* was expressed at parental level (pMV306-e*thR* construct) in the *ethA/R* KO Erdman strain (D).

**
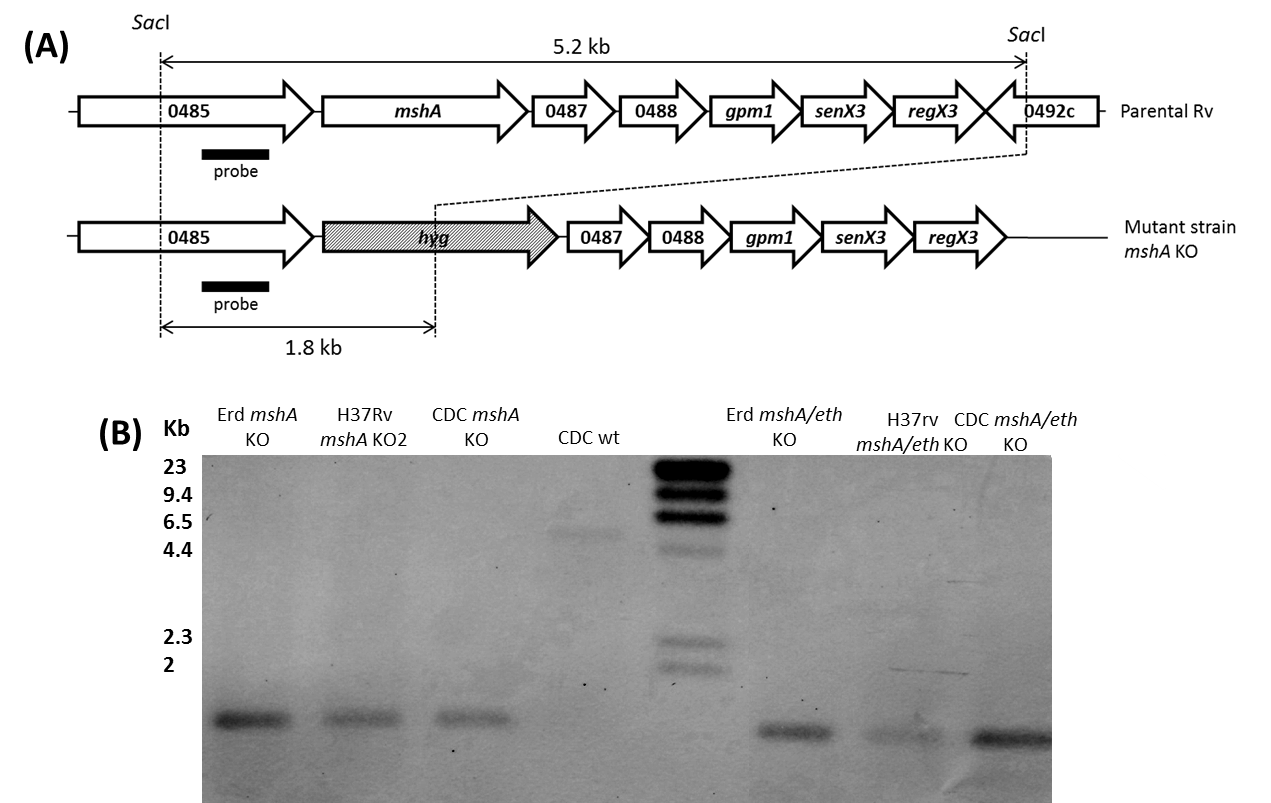
**

**Figure S5: Construction of *mshA* KO and *mshA/ethA/R* double KO mutants in MTB Erdman, MTB H37RV and MTB CDC1551**

A) Chromosomal organization of *mshA* was identical in the three MTB backgrounds. The arrows depict the lengths and directions of *mshA* and its neighbouring genes. Black bar corresponds to the probe used for Southern Blot analysis. B) Southern Blot analysis of chromosomal DNA. 1, MTB Erdman *mshA* KO; 2, MTB H37Rv *mshA* KO; 3, MTB CDC1551 *mshA* KO; 4, MTB CDC1551 WT ; L, DNA Molecular Ladder; 5, MTB Erdman *mshA/ethA/R* double KO; 6, MTB H37Rv *mshA/ethA/R* double KO; 3, MTB CDC1551 *mshA/ethA/R* double KO.

**Figure S6: ETH MIC curves on *mshA* KO and *mshA ethA/R* *(m/e)* double KO mutants.**

Drug susceptibility assay was performed in 7H9-OADC medium as described in M&M. OD600 values were read after 7 days incubation.

**Table S1: Primers used in this study.**

FP: Forward primer; RP: Reverse primer. RE: Restriction Enzyme. RE sites in primer sequences are underlined.

| **ETH^R^ Isolate** | **Gene Name / *Gene*** | **Known Function** | **Type of Mutations** | **No. of mutations** |
| --- | --- | --- | --- | --- |
| **760C1** | Erdman_0718 / *fabD2* | Malonyl CoA-acyl carrier protein transacylase | NS- SNPs | 1 |
|  | **Erdman_3212 / -** | **Malonyl CoA-acyl carrier protein transacylase** | **NS- SNPs** | **2** |
|  | Erdman_3280 / *gltx* | Glutamyl-tRNA synthetase | NS- SNPs | 1 |
| **760C3** | Erdman_0263 / - | Transmembrane protein | NS- SNPs | 1 |
|  | Erdman_0324 / - | Transmembrane protein | NS- SNPs | 1 |
|  | **Erdman_0532 / *mshA*** | **Mannosyl transferase** | **NS- SNPs** | **1** |
|  | Erdman_0693 / *recD* | Exonuclease V alpha chain | NS- SNPs | 1 |
|  | Erdman_1821 / *argB* | Acetylglutamate kinase | NS- SNPs | 1 |
|  | Erdman_3922 / - | Transcriptional regulator | NS- SNPs | 1 |
| **760C5** | **Erdman_0532 / *mshA*** | **Mannosyl transferase** | **NS- SNPs** | **1** |
|  | Erdman_0300 / *fadE6* | Acyl-CoA dehydrogenase | NS- SNPs | 1 |
|  | Erdman_0324 / - | Transmembrane protein | NS- SNPs | 1 |
|  | Erdman_0588 / *galE3* | UDP-glucose 4-epimerase | NS- SNPs | 1 |
|  | Erdman_1484 / - | Thioredoxin | NS- SNPs | 1 |
|  | Erdman_2261 / *pks12* | Polyketide synthase | NS- SNPs | 1 |
|  | Erdman_2922 / *-* | Prophage protein | NS- SNPs | 1 |
|  | **Erdman_3212 / -** | **Malonyl CoA-acyl carrier protein transacylase** | **NS- SNPs** | **1** |
|  | Erdman_3794 / *-* | REP13E12 repeat-containing protein | NS- SNPs | 1 |
| **770A1** | **Erdman_0532 / *mshA*** | **Mannosyl transferase** | **NS- SNPs** | **1** |
|  | Erdman_0819 / *-* | Transcriptional regulator | NS- SNPs | 1 |
|  | Erdman_1488 / *glgP* | Glycogen phosphorylase | NS- SNPs | 1 |
|  | Erdman_2377 / *murE* | UDP-N-acetylmuramoylalanyl-D-glutamate-2,6-diaminopimelate ligase | NS- SNPs | 1 |
|  | Erdman_2459 / *cobD* | Cobalamin biosynthesis protein | NS- SNPs | 1 |
|  | Erdman_2997 / *recX* | Recombination regulator | NS- SNPs | 1 |
|  | **Erdman_3212 / -** | **Malonyl CoA-acyl carrier protein transacylase** | **NS- SNPs** | **2** |
|  | Erdman_3992 / *topA* | DNA topoisomerase I | NS- SNPs | 1 |
| **770A3** | **Erdman_0532 / *mshA*** | **Mannosyl transferase** | **NS- SNPs** | **1** |
|  | Erdman_1118 / *pabB* | Para-aminobenzoate synthase component I | NS- SNPs | 1 |
|  | Erdman_1647 / *moxR1* | Transcriptional regulator | NS- SNPs | 1 |
|  | Erdman_1703 / *pks5* | Polyketide synthase | NS- SNPs | 1 |
|  | Erdman_2580 / *plcB* | Membrane-associated phospholipase C | NS- SNPs | 1 |
|  | **Erdman_3212 / -** | **Malonyl CoA-acyl carrier protein transacylase** | **NS- SNPs** | **1** |
|  | Erdman_4088 / *ligC* | ATP-dependent DNA ligase | NS- SNPs | 1 |
|  | Erdman_4115 / *tyrA* | Prephenate dehydrogenase | NS- SNPs | 1 |
| **60C72** | **Erdman_0532 / *mshA*** | **Mannosyl transferase** | **INDEL** | **1** |
| **80C73** | **Erdman_0532 / *mshA*** | **Mannosyl transferase** | **INDEL** | **1** |

**Table S2: Mutations identified in spontaneous ETH-resistant mutants.**

Spontaneous ETH-resistant mutants were derived from Erdman *ethA/R* KO mutant, screened for wildtype *inhA* gene, and 7 individual mutants were fully sequenced to identify novel genes involved in ETH bio-activation. Identified mutations were restricted to INDELs and NS-SNPs. The gene list was further refined by eliminating conservative NS-SNPs. Genes in bold are highlighted to reflect recurring (but random) mutations in selected mutants. INDEL – Insertion/Deletion, NS-SNPs – non-synonymous single nucleotide polymorphisms. Gene name, gene and known function were referenced and extracted from Pubmed Genbank. (<http://www.ncbi.nlm.nih.gov/nuccore/379026087>).

|  | **7H9-ADS** | **7H9-ADS** | **7H9-OADC** | **7H9-OADC** |
| --- | --- | --- | --- | --- |
|  | INH | ETH | INH | ETH |
| **CDC1551** | 0.22 | 6.90 | 0.26 | 7.18 |
| **CDC1551 *ethA/R* KO** | 0.22 | 12.40 | 0.20 | 31.04 |
| **Erdman** | 0.15 | 3.89 | 0.18 | 3.04 |
| **CDC1551 *ethA/R* KO** | 0.11 | 9.29 | 0.18 | 19.69 |
| **H37Rv** | 0.20 | 3.30 | 0.20 | 3.30 |
| **H37Rv *ethA/R* KO** | 0.20 | 9.16 | 0.28 | 12.55 |

**Table S3: MIC_50_ values of INH and ETH in 7H9-ADS and 7H9-OADC in parental and *ethA/R* KO strains.**

Drug assays were set up in 96-well plates using a broth microdilution method and their OD600 values were read with a spectrophotometry microplate reader after 5-7 days. OD600 values were tabulated into PRISM for fitting of MIC curves, and MIC_50_ values were read from PRISM.
